# Effects of aerobic exercise training on muscle plasticity in a mouse model of cervical spinal cord injury

**DOI:** 10.1038/s41598-020-80478-9

**Published:** 2021-01-08

**Authors:** Isley Jesus, Pauline Michel-Flutot, Therese B. Deramaudt, Alexia Paucard, Valentin Vanhee, Stéphane Vinit, Marcel Bonay

**Affiliations:** 1 Inserm, END-ICAP, Université Paris-Saclay, UVSQ, 78000 Versailles, France; 2 Service de Physiologie-Explorations Fonctionnelles; Hôpital Ambroise Paré, Assistance Publique-Hôpitaux de Paris, Boulogne, France

**Keywords:** Cell biology, Physiology, Diseases

## Abstract

Cervical spinal cord injury (SCI) results in permanent life-altering motor and respiratory deficits. Other than mechanical ventilation for respiratory insufficiency secondary to cervical SCI, effective treatments are lacking and the development of animal models to explore new therapeutic strategies are needed. The aim of this work was to demonstrate the feasibility of using a mouse model of partial cervical spinal hemisection at the second cervical metameric segment (C2) to investigate the impact of 6 weeks training on forced exercise wheel system on locomotor/respiratory plasticity muscles. To measure run capacity locomotor and respiratory functions, incremental exercise tests and diaphragmatic electromyography were done. In addition, muscle fiber type composition and capillary distribution were assessed at 51 days following chronic C2 injury in diaphragm, extensor digitorum communis (EDC), tibialis anterior (TA) and soleus (SOL) muscles. Six-week exercise training increased the running capacity of trained SCI mice. Fiber type composition in EDC, TA and SOL muscles was not modified by our protocol of exercise. The vascularization was increased in all muscle limbs in SCI trained group. No increase in diaphragmatic electromyography amplitude of the diaphragm muscle on the side of SCI was observed, while the contraction duration was significantly decreased in sedentary group compared to trained group. Cross-sectional area of type IIa myofiber in the contralateral diaphragm side of SCI was smaller in trained group. Fiber type distribution between contralateral and ipsilateral diaphragm in SCI sedentary group was affected, while no difference was observed in trained group. In addition, the vascularization of the diaphragm side contralateral to SCI was increased in trained group. All these results suggest an increase in fatigue resistance and a contribution to the running capacity in SCI trained group. Our exercise protocol could be a promising non-invasive strategy to sustain locomotor and respiratory muscle plasticity following SCI.

## Introduction

A spinal cord injury (SCI) results in permanent life-altering neuromotor functions due to the limited recovery capacity of the central nervous system. When the injury is located at the cervical level, sensory-motor functions below the site of lesion are lost and permanent respiratory insufficiency is induced^1,2^. Rat models are widely used to study SCI^3^, while mouse models of cervical injury are only used to assess post-traumatic spontaneous locomotor and respiratory muscle recovery^3–5^. It is clear that molecular studies are required to better comprehend the mechanisms involved in cervical SCI. Since rat SCI models are limited by the poor availability of genetically modified specimens, mouse models, with an extensive number of genetically modified strains may be a preferable option to decipher the intricacy of molecular and cellular interactions^6,7^. Generally, the recovery of the diaphragm after SCI is quite limited^8–10^. Our laboratory has recently shown that diaphragm recovery remained significantly reduced 30 days after injury in a mouse model of cervical SCI^11^. In fact, the diaphragm is the major muscle responsible for breathing and also participates in non-ventilatory behaviors associated with airways clearance, such as coughing and sneezing^12–14^. Moreover, SCI injury impacts other components of the diaphragm motor unit, mainly at the level of the neuromuscular junction and the muscle fiber types and size^9^. At day 1, a reduction in ipsilateral diaphragm cross-sectional area of type I and IIa myofibers is observed, while type IIb/x myofibers have reduced cross-sectional area at both 1 and 7 days following SCI in rat model^15^. At 42 days, the size of type IIb/x myofibers is reduced, and hypertrophy is no longer evident in type I or IIa myofibers^9^. These previous findings suggest that rats with SCI present a tremendous capability of muscular remodeling following injury. In addition, impairment and paralysis of skeletal muscles that are innervated caudal to the lesion site are accompanied by a shift from an oxidative to a highly glycolytic, and fatigable phenotype in both animals and humans^15–17^. Furthermore, mitochondrial capacity of skeletal muscles is critically reduced after SCI and supply undergoes remodeling, typically in the form of capillary rarefaction in the muscle^18^. This rarefaction impairs the capacity to deliver oxygen and fuel to working muscles, contributing to reduced resistance to fatigue^19^.

The literature has well established that exercise enhances functional recovery following injury in rodents^20–22^. Recently, Gorgey et al. showed that electrical stimulation-evoked resistance training (mimicking motor demands) combined with testosterone replacement therapy resulted in fiber hypertrophy and beneficial changes in markers of skeletal muscle health and function in persons with SCI^23^. The stimulation of fore/hindlimb muscles by rehabilitation exercise is used as a strategy to enhance stepping ability and respiratory recovery post SCI in both animal models and clinical studies^20,22,24^.These evidences reinforce the hypothesis that stimulation of limb muscles may be useful to improve respiratory rehabilitation post SCI. Nevertheless, little is known about the microvascular modulation and the effect of a non-invasive intervention (i.e. exercise training paradigm) on diaphragm morphology post-SCI. To date, there is no study on SCI mouse model that investigates the role of a long-term exercise training paradigm on locomotor and respiratory muscle plasticity. The most used behavior assessment qualitative tests are the Basso, Beattie and Bresnahan score (BBB score) for rodent, and the BMS (Basso Mouse Scale) for mice. These tests are generally performed to evaluate locomotor recovery^25–27^. However, incremental exercise test is largely used in both healthy animals models and humans to evaluate running capacity, but has not yet been used to evaluate the performance exercise after spinal cord injury^28–31^. Therefore, the purpose of this work was to demonstrate the feasibility of using a mouse model of partial cervical spinal hemisection at the second cervical metameric segment (C2) to investigate the impact of 6 weeks training on forced exercise wheel system on locomotor/respiratory plasticity muscles in our mouse model. We aimed: (1) to evaluate the animal running capacity (at different post-lesion time points) on forced exercise wheel system, (2) to investigate the effect of SCI on locomotor forelimb [extensor digitorum communis (EDC)], hindlimb [Tibialis anterior (TA) and soleus (SOL)] and respiratory (DIA) muscles morphology and microcirculation following 6 weeks of low intensity exercise training paradigm. Diaphragmatic electromyography was also recorded at the end of the training protocol to determine the recovery of the diaphragm activity.

## Results

### Maximal physical capacity

**Figure 1:**
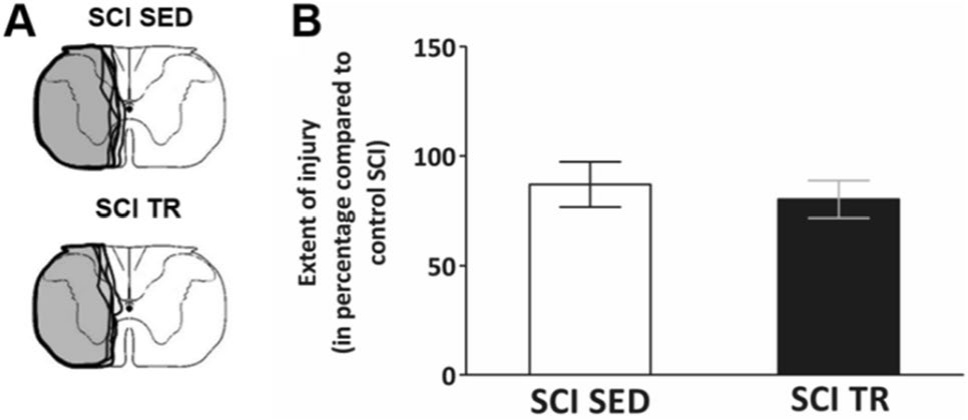
Extent of injury following a C2 hemisection. (**A**) Schematic representation of the extent of injury for each animal in SCI SED and SCI TR groups. (**B**) Graph shows the quantification of the extent of injury in percentage compared to a 100% hemisected spinal cord. The quantification has only been made in the ventral part where the phrenic motoneurons are located. Data are presented as mean ± SD (n = 6). There is no difference between the different groups (t-test, *p* = 0.4127).

The size of the cervical SCI lesion was similar in the lateral part of both groups of injured animals (SCI sedentary (SED) and SCI trained (TR) groups) (Fig. 1A). Quantitatively, no significant difference in the size of injury between SCI SED and SCI TR groups was observed (86.99 ± 10.3% and 80.23 ± 8.5%, respectively, *p* = 0.329) (Fig. 1B).

**Figure 2:**
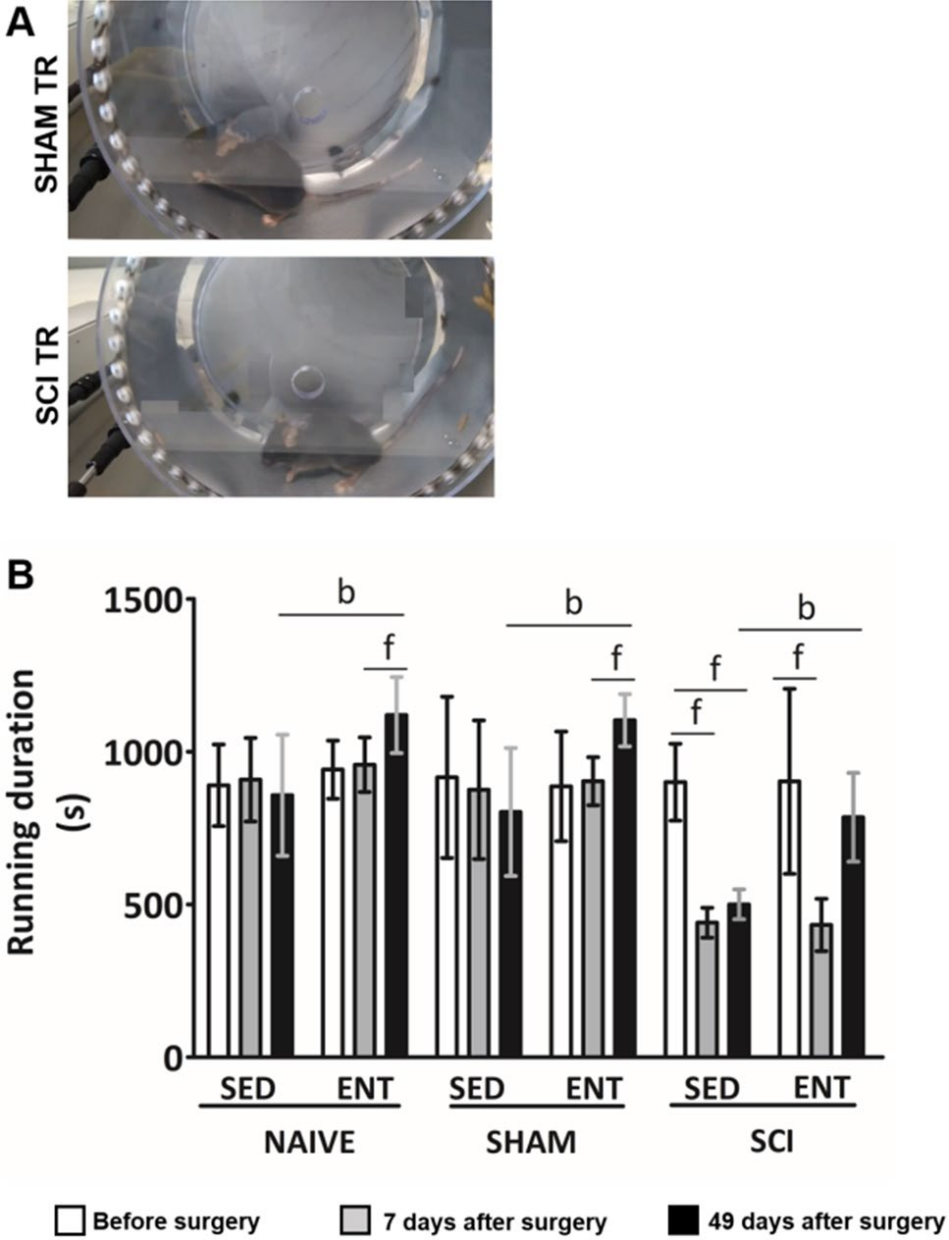
Maximal physical capacity expressed as running time (s). (**A**) Representative snapshot of a SHAM (upper) and SCI (below) mouse running in forced exercise wheel. (**B**) Running capacity on forced exercise wheel at different time points (before SCI; 7 days after surgery and 49 days after surgery). Data are presented as mean ± SD (n = 6). Significance: b *p* < 0.05 compared to sedentary, f *p* < 0.05 compared to before surgery or 7 days after surgery.

After 6 weeks of training on the forced exercise wheels (Fig. 2A), running capacity, which was expressed as running time (s), was higher in all trained groups (Fig. 2B).

### Morphometric analysis of locomotor muscle

**Figure 3:**
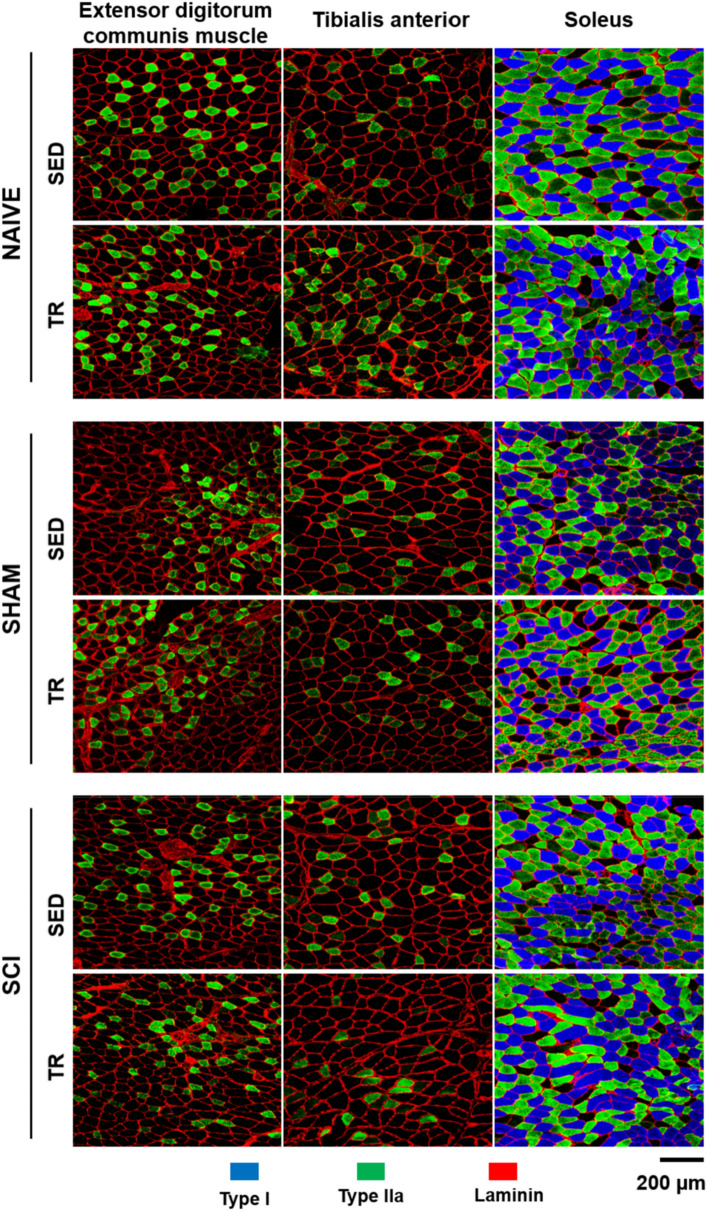
Representative images of fiber type composition in locomotor muscles and analysis of fiber type composition. Extensor digitorum communis (EDC), Tibialis anterior (TA) and Soleus (SOL) cryosections were immunostained to identify Myosin heavy chain (MyHC) isoforms: MyHCSlow (type I in blue), MyHC2A (type IIa in green) co-labeled with an anti-laminin antibody to visualize myofiber boundaries (type IIb/x corresponding to unlabeled fibers). Scale bar = 200 μm.

To investigate whether forced exercise wheel training alters the muscle fiber composition in SCI mice, MyHC isoforms (MyHCSlow = type I, MyHC2A = type IIa and non-labeling = IIb/x) were detected by immunofluorescence staining (Fig. 3). Although soleus muscle alone presented a type I staining, the predominant types of myofibers in the ipsilateral forelimb (EDC) and hindlimb (TA and SOL) muscles of trained and sedentary animals were compared as well (Fig. 3). The morphometric results were described in Table 1 for SOL muscle, while those of EDC and TA muscles were added as supplemental data 1.

**Table 1:** Soleus muscle fiber type area and distribution.

	NAIVE		
	SED	TR	*p* value
**Area. μm**^**2**^			
Type I	1613.3 ± 389.3	1434.3 ± 249.4	0.242
Type IIa	1088.3 ± 329.2	926.2 ± 240.4	0.281
Type IIb/x%	902.8 ± 166.9	803.8 ± 274.7	0.36
**%**			
Type I%	45.2 ± 7.7	44.4 ± 8.7	0.877
Type IIa%	42.3 ± 10.0	42.9 ± 7.9	0.907
Type IIb/x%	12.4 ± 2.8	12.5 ± 5.2	0.957

	SHAM		
	SED	TR	*p* value
**Area. μm**^**2**^			
Type I	1303.9 ± 115.0	1081.5 ± 266.0	0.149
Type IIa	743.5 ± 158.8	622.4 ± 291.8	0.418
Type IIb/x%	538.9 ± 138.2	645.0 ± 185.1	0.224
**%**			
Type I%	55.7 ± 4.9	51.4 ± 11.0	0.382
Type IIa%	37.0 ± 8.6	35.7 ± 10.0	0.786
Type IIb/x%	7.2 ± 4.0	12.8 ± 7.8	0.078

	SCI		
	SED	TR	*p* value
**Area. μm**^**2**^			
Type I	1336.0 ± 222.7	1453.6 ± 239.3	0.439
Type IIa	837.3 ± 164.3	833.0 ± 297.3	0.977
Type IIb/x%	716.7 ± 176.4	756.8 ± 221.9	0.816
**%**			
Type I%	45.3 ± 5.2	46.2 ± 7.9	0.818
Type IIa%	37.7 ± 10.1	38.9 ± 12.2	0.853
Type IIb/x%	16.9 ± 7.0	14.7 ± 6.0	0.582

Values are means ± SD for NAIVE, SHAM and SCI (SED and TR).

### Vascularization analysis of locomotor muscle

**Figure 4:**
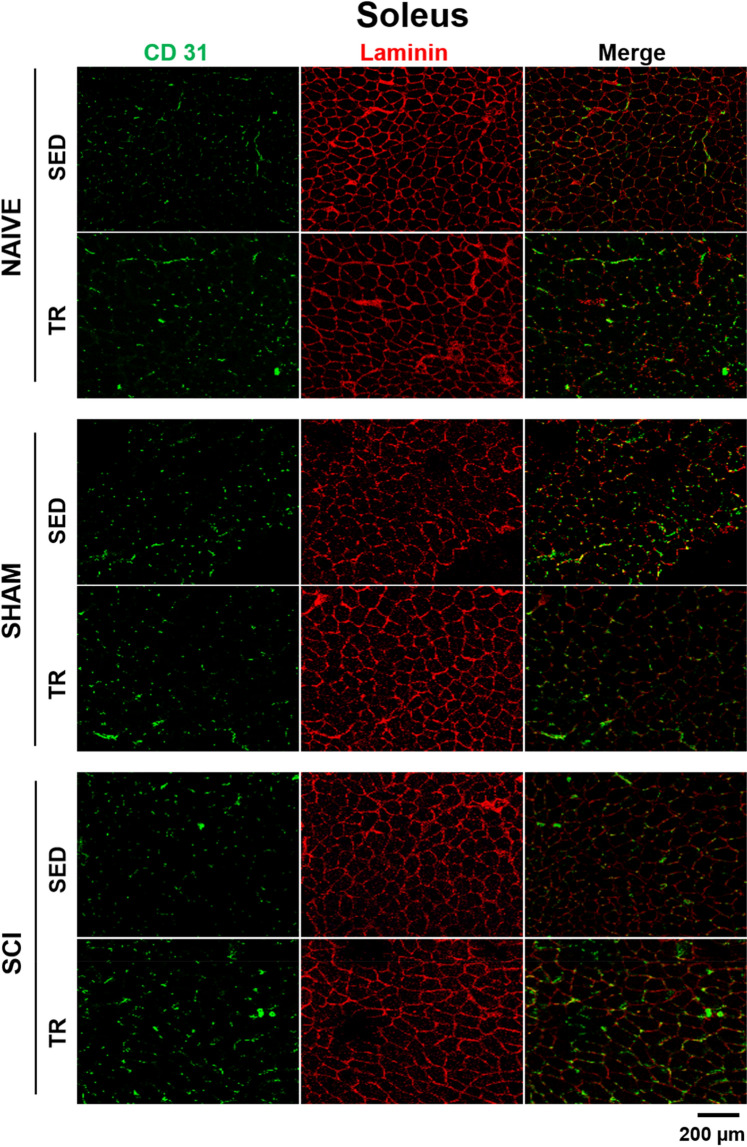
Representative images of CD31 expression and number of fibers in the skeletal muscles. Cryosections were immunostained to visualize CD31 (green) co-labeled with an anti-Laminin antibody (in red) in Soleus (SOL), while Extensor digitorum communis (EDC), Tibialis (TA) anterior are illustrated in supplemental Figs. 1 and 2, respectively. Scale bar = 200 μm.

To assess the capillary response to aerobic exercise, cryosections were immunostained to visualize CD31 (green) co-labeled with an anti-Laminin antibody (in red) in SOL (Fig. 4), while EDC and TA are found in supplementary Fig. 1. Capillary density (CD) and capillary-to-fiber ratio were analyzed and described in the Table 2 for SOL while EDC and TA are in supplementary Table 2. Our data showed that 6 weeks of exercise training increased the number of muscle capillaries in the hindlimb (TA and SOL) of mice from SCI group. Capillary density and capillary-to-fiber ratio in TA and SOL muscles were increased in SCI TR group compared to those of SCI SED group. In addition, the capillary density in TA muscle was higher in SCI TR group than in NAIVE TR group. The same tendency was observed in the SOL muscle. In SCI SED group, the capillary density of TA muscle was significantly lower than that seen in NAIVE SED group.

**Table 2:** Soleus muscle capillarization.

	SED	TR	*p* value
NAIVE			
CD/mm ^2^	1473.1 ± 839.2	1319.2 ± 372.4	0.972
Capillaries/fiber	1.1 ± 0.4	1.6 ± 0.4	0.112
SHAM			
CD/mm ^2^	1113.8 ± 400.6	1777.2 ± 625.0	0.058
Capillaries/fiber	1.2 ± 0.4	1.5 ± 0.4	0.35
SCI			
CD/mm ^2^	1685.3 ± 481.0	2655.9 ± 649.6**	0.007
Capillaries/fiber	1.3 ± 0.4	2.3 ± 0.8**	0.003

Values are means ± SD for NAIVE, SHAM and SCI (SED and TR). Training effect between SED and TR groups **p < 0.01.

### SCI effect on diaphragm activity following exercise training

**Figure 5:**
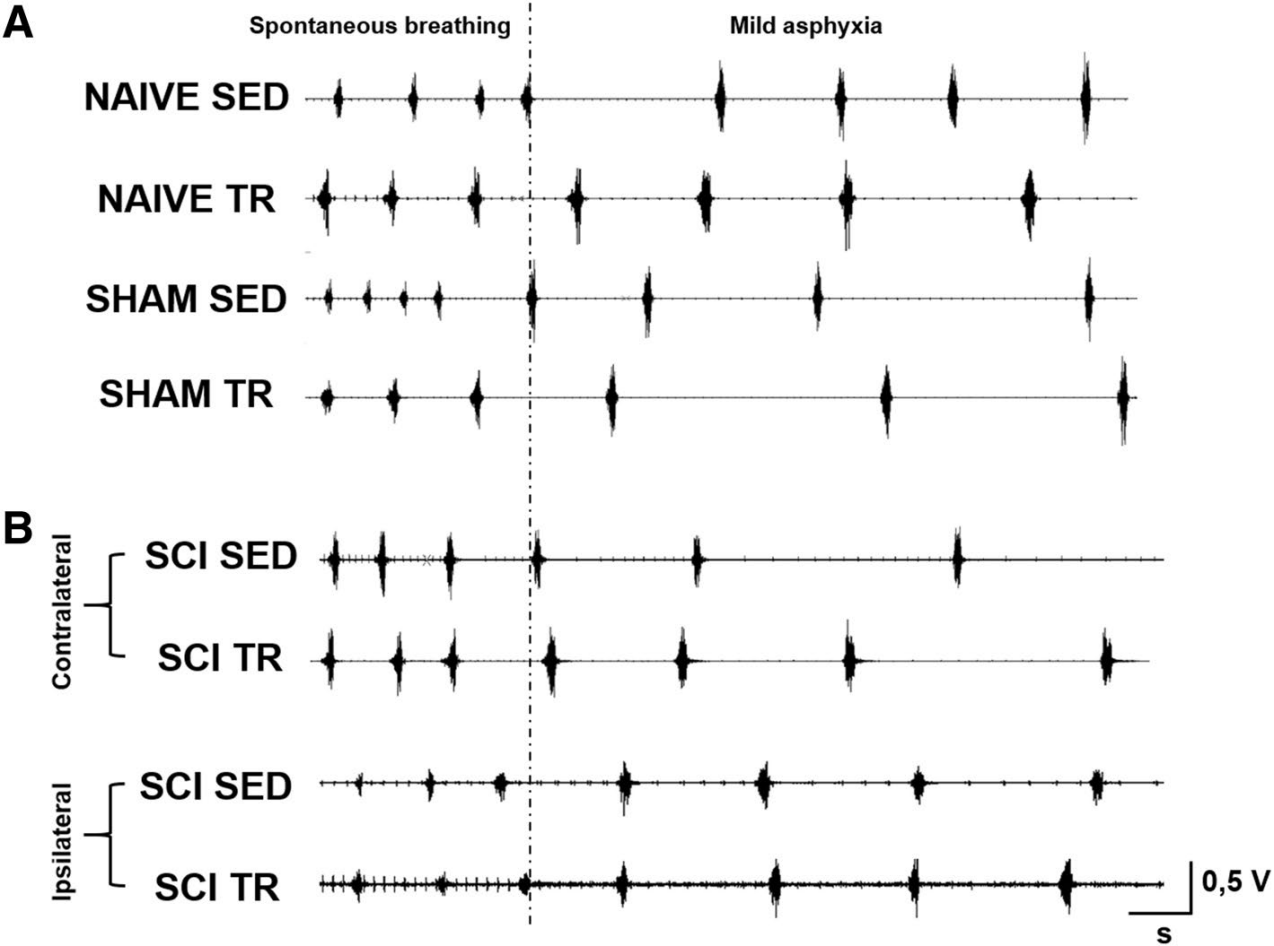
Diaphragm activity at 51 days following a C2 hemisection. (**A**) Representative traces of diaphragm raw electromyography for uninjured animals: NAIVE and SHAM groups (SED and TR) and (**B**) injured (contralateral diaphragm and ipsilateral diaphragm) animals: SCI groups (SED and TR).

Representative traces of raw diaphragm electromyography at 51 days following SCI are shown in Fig. 5A (uninjured (NAIVE /SHAM, SED and TR) and injured animals (SCI, contralateral and ipsilateral DIA, SED and TR) in Fig. 5B.

**Figure 6:**
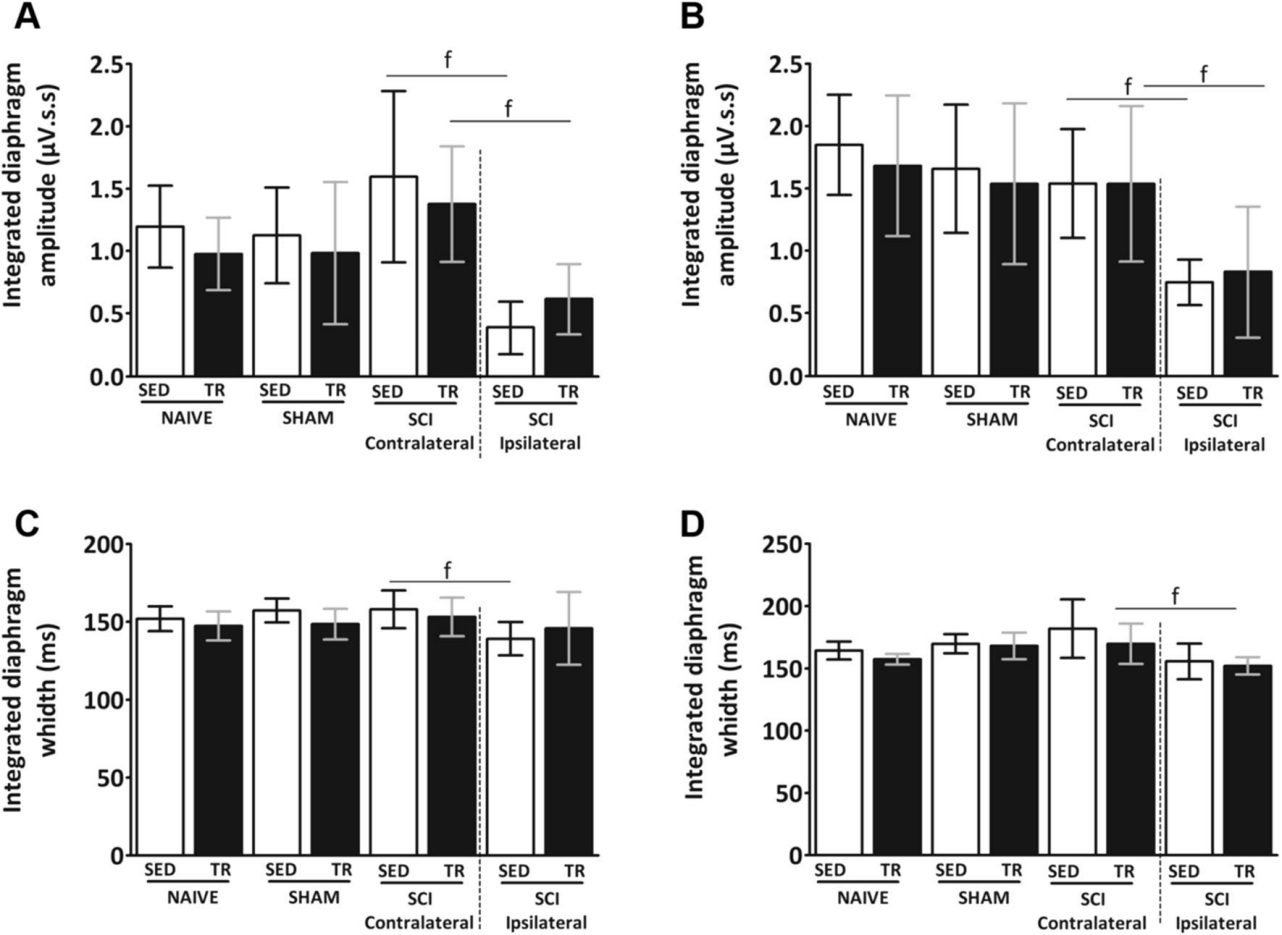
Diaphragm activity analysis at 51 days following a C2 hemisection. Graphs show integrated diaphragm amplitude during (**A**) spontaneous breathing and (**B**) mild asphyxia. Graphs represent the width from each EMG bursts of integrated diaphragm signal during (**C**) spontaneous breathing and (**D**) mild asphyxia. Data are presented as mean ± SD (n = 6). Significance: f *p* < 0.05 compared to ipsilateral side.

A reduced diaphragm electromyography activity during spontaneous breathing and mild asphyxia on the ipsilateral side in SCI groups was observed when compared to the contralateral side. Diaphragm electromyography during spontaneous breathing and mild asphyxia did not show any difference in amplitude on the contralateral side of injured and uninjured animals groups. Quantitatively, uninjured animals groups showed a similar integrated diaphragm average amplitude (Fig. 6A,B) compared to the contralateral side of the SCI SED. Fifty-one days following SCI, the integrated diaphragm average amplitude during spontaneous breathing and mild asphyxia on the ipsilateral side was reduced compared to the contralateral side. The contraction time was measured from the width of each EMG bursts respiration of animal. No significant difference was observed during spontaneous breathing. The width from each EMG bursts on the ipsilateral side was significantly decreased during spontaneous breathing (Fig. 6C) was observed in SCI SED group when compared to the contralateral side, while in the SCI TR group no difference was observed between the two diaphragm sides. Interestingly, a decrease in the duration of integrated average electromyography during mild asphyxia (Fig. 6D) was observed in the SCI TR group.

### Morphometric analyses of respiratory muscle

**Figure 7:**
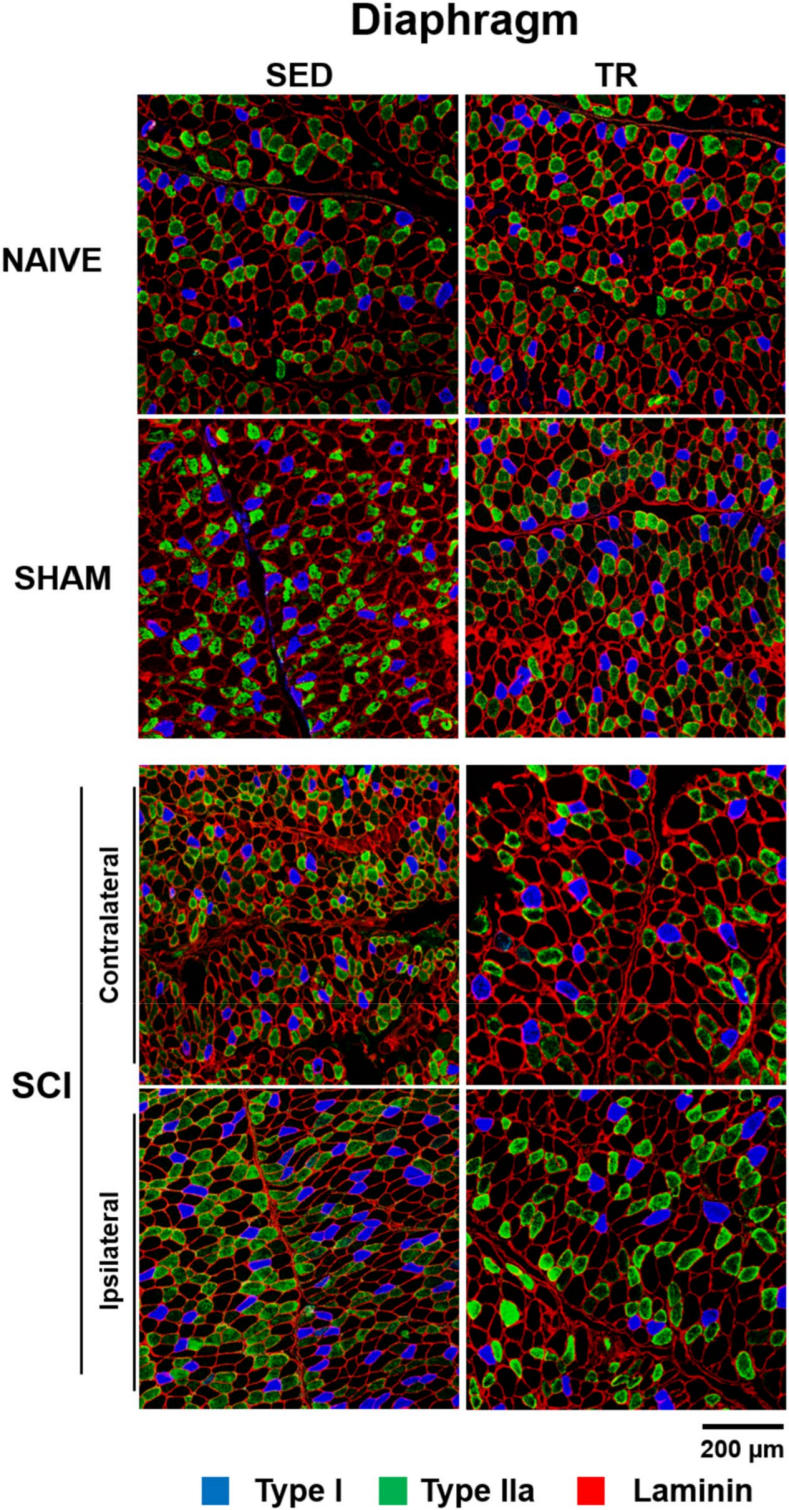
Representative images of fiber type composition in respiratory muscle. Diaphragmatic muscle cryosections were immunostained to visualize Myosin heavy chain (MyHC) isoforms: MyHCSlow (type I in blue), MyHC2A (type IIa in green) co-labeled with an anti-laminin antibody to visualize myofiber boundaries (type IIb/x corresponding to unlabeled fibers). Scale bar = 200 μm.

To investigate the effect of our forced exercise wheel training protocol in respiratory muscle recovery following SCI, MyHC isoforms (MHC I, IIa and negative label IIb/x) were detected by immunofluorescence staining (Fig. 7).

**Figure 8:**
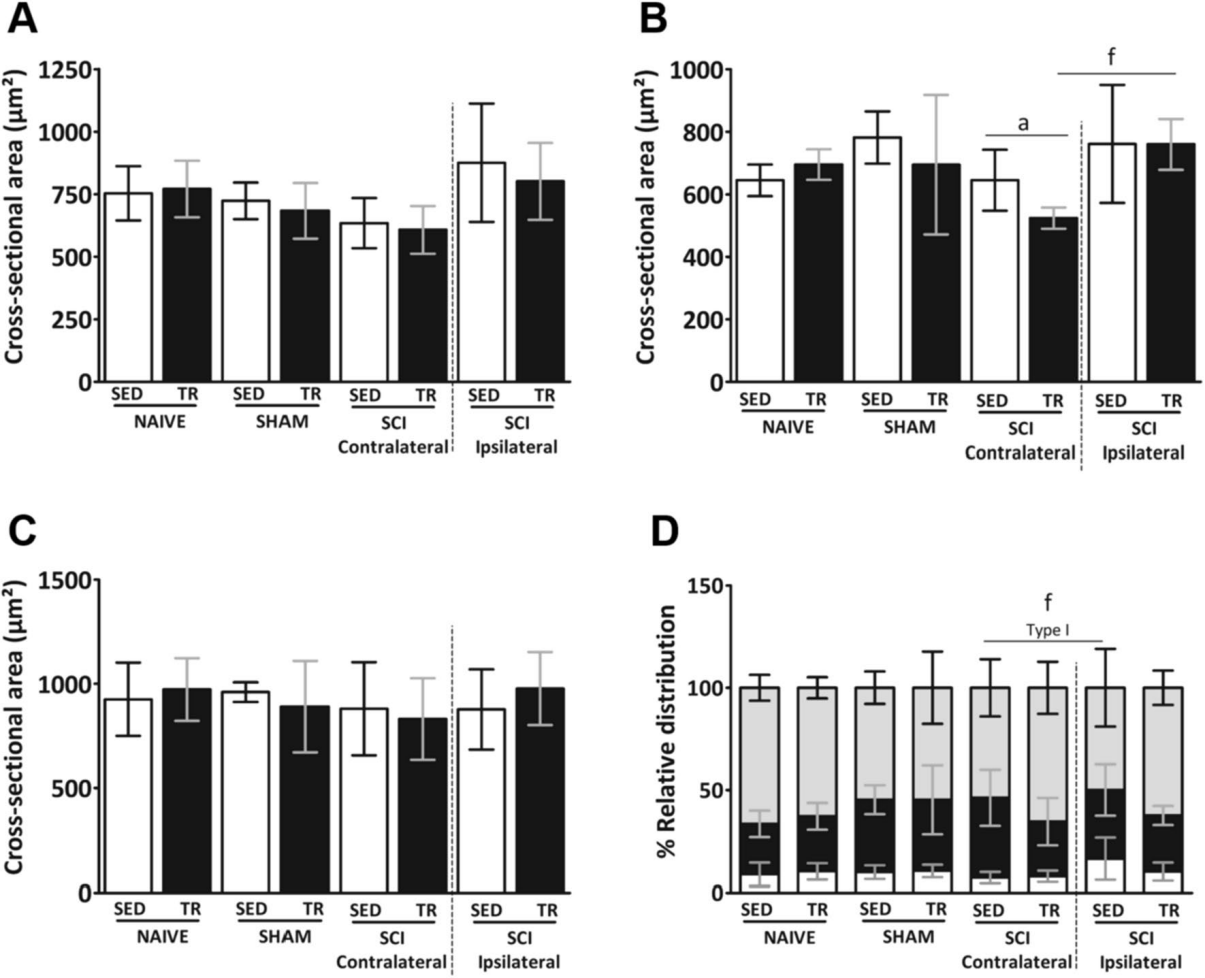
Analysis of fiber type composition in the respiratory muscle. (**A–C**) muscle fiber cross sectional area and (**D**) percentage of fiber type relative in diaphragmatic muscle. Data are presented as mean ± SD (n = 6). Significance: a *p* < 0.05 compared to TR group; f *p* < 0.05.

The predominant fiber types in the contralateral and ipsilateral diaphragm muscle were analyzed (Fig. 8A–D). In cross-sectional area of type I myofiber staining (Fig. 8A), no significant increase was observed on the ipsilateral side of both SCI groups (SED and TR) compared to their respective contralateral side. Analysis of the cross-sectional area on the contralateral side of the SCI TR group showed a decrease in type IIa myofiber when compared to its ipsilateral side and to the contralateral side of SCI SED group (Fig. 8B). In the cross-sectional area stained for type IIb/x myofiber (Fig. 8C), no difference was observed between groups or between diaphragm sides. Relative distribution (Fig. 8D) was altered between the contralateral and the ipsilateral sides in SCI SED group, while no difference was observed in the SCI TR group.

### Vascularization analysis of respiratory muscle

**Figure 9:**
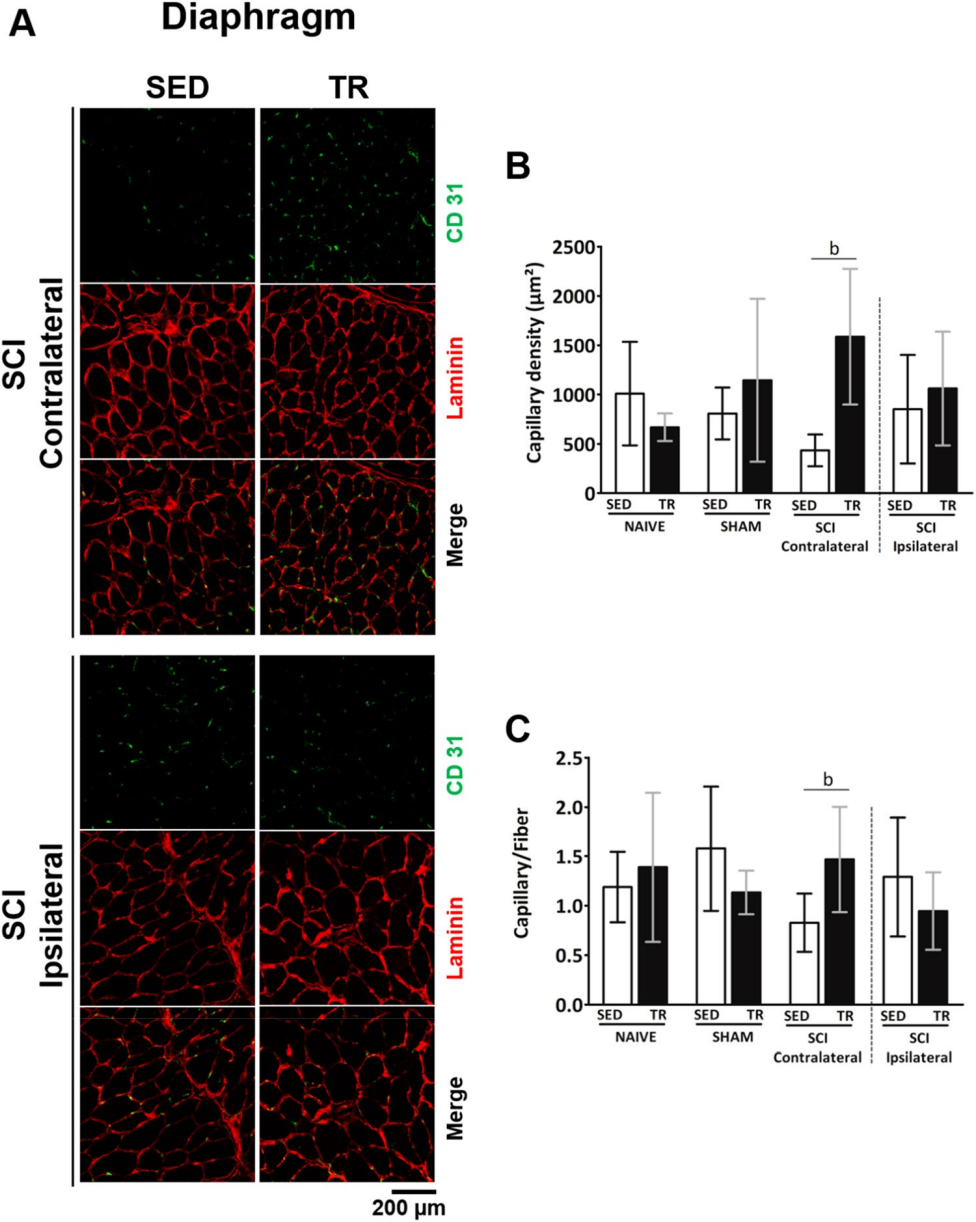
Analysis of endothelial cells on respiratory muscle. (**A**). Cryosections were immunostained to visualize CD31 (green) co-labeled with an anti-Laminin antibody (in red) in the diaphragm muscle. Scale bar = 200 μm. Comparison of CD31 expression and number of fibers in the diaphragm muscle represented by SED and TR SCI groups, while NAIVE and SHAM groups (SED and TR) are illustrated in supplemental Figs. 3. Scale bar = 200 μm. (**B**) Capillary density and (**C**) Ratio between capillary and fiber. Data are presented as mean ± SD (n = 6). Significance: b *p* < 0.05 compared to SED group.

To assess the capillary response to exercise, capillary density and capillary-to-fiber ratio in diaphragm muscle were evaluated by immunofluorescence (Figs. 9A and supplemental Fig. 3 for NAIVE and SHAM groups). Six weeks of exercise training increased the capillary density and capillary-to-fiber ratio (Figs. 9B,C) on the contralateral side of SCI group. However, no significant difference in the capillary density was observed between the ipsilateral side of SED and TR SCI groups.

## Discussion

Consistent with the possibility that exercise enhances locomotor recovery following SCI in mice, 6 weeks of exercise training on wheel running system effectively improved the running capacity in our mouse model. While our exercise protocol did not change the morphology of limb muscles, an increase in the microcirculation in the SCI TR group was observed. This current study showed for the first time that, even after 51 days, diaphragm recovery following SCI in mice is quite limited. Our running exercise training did not induce any effective recovery of the electromyography activity of impaired diaphragm in SCI mouse. The width of each EMG burst was reduced in SCI sedentary group during spontaneous breathing, while it remained similar in the SCI trained group and uninjured groups. During mild asphyxia, this reduction was only observed in TR SCI groups. Cross-sectional area of type I myofiber in the diaphragmatic muscle showed a slight increase on the ipsilateral diaphragm in both SED and TR SCI groups. Exercise training decreased type IIa myofiber on the contralateral side. Moreover, we observed that 6 weeks of aerobic exercise training increased vascularization in the intact diaphragm of SCI TR group compared to SCI SED group.

Cervical SCI commonly affects ipsilateral forelimb and rehabilitative training is usually provided for the recovery of locomotor skills^32,33^. Usually, BBB score is widely used to test behavioral consequences of training efficacy in animal models of spinal cord injury^26,27^. In a previously published study, a rat model of cervical contusion performed an 8-week exercise training in a forced wheel system^22^. In this work, the speed was increased daily according to the abilities of each animal up to 14 m/min and the BBB scale was used to evaluate locomotor skill. We chose a semi-quantitative test as a strategy to assess running capacity by using an incremental test. Several studies have used incremental exercise capacity in both animal models and humans as a strategy to evaluate performance after a prolonged exercise training^28–31^. Since running capacity has not yet been used in mice following chronic SCI, we thought it relevant to test it in our work. One of our aims was to show that incremental exercise capacity test could be used in addition to other well established behavioral assessment methods to obtain more information on locomotor recovery following spinal cord injury. Therefore, seven days after cervical SCI, mice presented a running capacity significantly reduced. On the other hand, a 6-week exercise training (49 days post SCI) induced a running capacity recovery similar to the level observed pre-SCI.

In rodent models, SCI impacts dramatically the muscle morphology within the first weeks following injury^34,35^. However, one week of training can efficiently lead to an increase in the regenerative process of slow-twitch muscle^36,37^. Few studies have assessed the effects of long periods of locomotor training on muscle properties in a mouse model of cervical hemisection SCI. Battistuzzo et al. have performed a treadmill-training paradigm on thoracic hemisected mice, with the speed initially set at 6 m/min and then, gradually increased to 10–12 m/min over the course of training. This protocol led to a slight increase in fiber cross-sectional area in SOL muscle at 10 weeks post-training^16^. In comparison to the prior training paradigm, we were surprised to observe no change in limbs muscle size. However, we do consider several parameters which could explain these negative results. Firstly, there is no published work linking the effect of training with forced exercise wheel system on muscle size, since treadmill is usually used in this type of study. This suggests that due to the difference in environment between forced exercise wheel system and treadmill, more elevated exercise intensities may be required with the forced exercise wheel system for changes in muscle size to be observed. Secondly, the lack of modification in fiber cross-sectional area of skeletal muscle may be due to the exercise intensity or the training duration applied in this study. Usually, in prolonged endurance exercise in rodents, the intensity of exercise is adjusted to maintain the training intensity^31^. However, in this present study the exercise adjustment was not done in order to prevent any bias in our procedure between the uninjured and injured animals.

Despite the ineffectiveness of our exercise training protocol to remodel the fiber cross-sectional area of the limb muscles, an increase in microcirculation in the SCI TR group was observed. Contrary to skeletal muscles, microvascular supply undergoes remodeling, typically in the form of capillary rarefaction after SCI^19^. The capacity to deliver oxygen and fuel to the working muscles is de facto impaired and contributes to reduce fatigue resistance^18,38^. Moreover, a capillary rarefaction could contribute to the impairment of insulin sensitivity and glucose intolerance which may be further aggravated by systemic inflammation^39^ and posttraumatic inflammatory response in SCI secondary pathogenesis^40,41^.

Locomotor intervention is the most suitable procedure for the rehabilitation of person with SCI. Aerobic exercise training is well established in the literature and is described as beneficial towards improvement in aerobic capacity and increase in mitochondrial density as well as increase in citrate synthase^42,43^. Prolonged endurance exercise training leads to an increase in skeletal muscle microcirculation^31^, and based on observation in rodents, it has been suggested that the rate of microvessel proliferation (angiogenesis) is faster in response to low intensity aerobic exercise training^44^. This could be beneficial to provide a better structure in a diaphragm morphology following SCI. From this standpoint, we hypothesized that direct inputs to the respiratory from the mesencephalic locomotor region may provide an interaction between spinal locomotor network and brainstem respiratory centers^16,20,21,45^. However, the mechanisms involved remain unclear^20^. In this study, we investigated the modulation of diaphragm activity in SCI mouse model following a 6-week exercise training protocol. Electromyography activity was recorded at the end of the exercise training to determine its effect on the recovery of diaphragm activity in the SCI mouse. Post-injury plasticity is attributed to the “crossed phrenic phenomenon” (CPP), which consists in the activation of silent pathways crossing the spinal cord midline at the C3-C6 segmental level and connects to the ipsilateral deafferented phrenic motoneurons^46,47^. However, it has been suggested that this spontaneous post-injury plasticity process, named, “spontaneous crossed phrenic phenomenon”, could be different from the initial pathways described as the CPP and could involve axonal sprouting and rerouting, and the formation of new polysynaptic connections to phrenic motoneurons via cervical spinal interneurons^48–51^. Nevertheless, the recovery of phrenic motor function remains limited and deficits in breathing persist for months post-injury^8,51^. With regard to the impact of SCI on diaphragm muscle, we investigated the effect of a putative non-invasive strategy to enhance long-term diaphragmatic plasticity and remodeling following SCI. Long-term study with rat models of cervical SCI has previously demonstrated that 42 days post-injury, diaphragm morphology exhibited a reduction in type IIb/x myofiber, and hypertrophy was no longer evident on type I or IIa myofibers^9^. In addition, reduction in contractile force occurs in parallel with altered cross-sectional area of fibers. In our study, cross sectional area of type I myofiber showed a slight increase in ipsilateral diaphragm in both SED and TR SCI groups. On the other hand, exercise training decreased type IIa myofiber in the contralateral side. Moreover, we investigated for the first time the effect of aerobic exercise on the induction of angiogenesis in the diaphragm of SCI mice. Therefore, the capillaries in the intact diaphragm of SCI TR group were increased compared to SCI SED group. As previously demonstrated, this increase in capillary density could contribute to the switch in fiber type in the limb muscles^52–54^. This suggests that morphology and vascular remodeling of the diaphragm contributed to avoid imbalance of fiber distribution between diaphragm sides. More experiments are required to investigate if the changes on diaphragm morphology and vascular structures in SCI trained group are correlated with diaphragm contraction duration analyzed by electromyography.

Based on these data, we showed that incremental test is a valuable method for evaluating the running capacity in mice with cervical spinal cord injury. Consequently, aerobic exercise training improves the microcirculation of locomotor muscles. In addition, our protocol showed a favorable role on diaphragm myofibers and vascularization after cervical spinal cord hemisection. We admit that 6 weeks of low exercise intensity are not enough to induce a recovery of the respiratory function after cervical spinal cord hemisection. Nevertheless, our data allow us to consider aerobic exercise training as a good non-invasive strategy to use in order to sustain locomotor and respiratory muscle plasticity following cervical SCI.

## Material and methods

### Ethics statement

All experiments reported in this manuscript were conformed to the policies laid out by the Guide for the Care and Use of Laboratory Animals in the EU Directive 2010/63/EU for animal experiments. These experiments were approved by the Ethics committee of the University of Versailles Saint-Quentin-en-Yvelines and complied with the French and European laws regarding animal experimentation (Apafis #2018011916127707v4). These experiments were performed on adult C57BL/6 male and female mice (8–10 weeks old). The animals were housed in individually ventilated cages in a state-of-the-art animal care facility (2CARE animal facility, accreditation A78-322-3, France), with access to food and water ad libitum on a 12 h light/dark cycle.

### Incremental exercise test protocol

**Figure 10:**
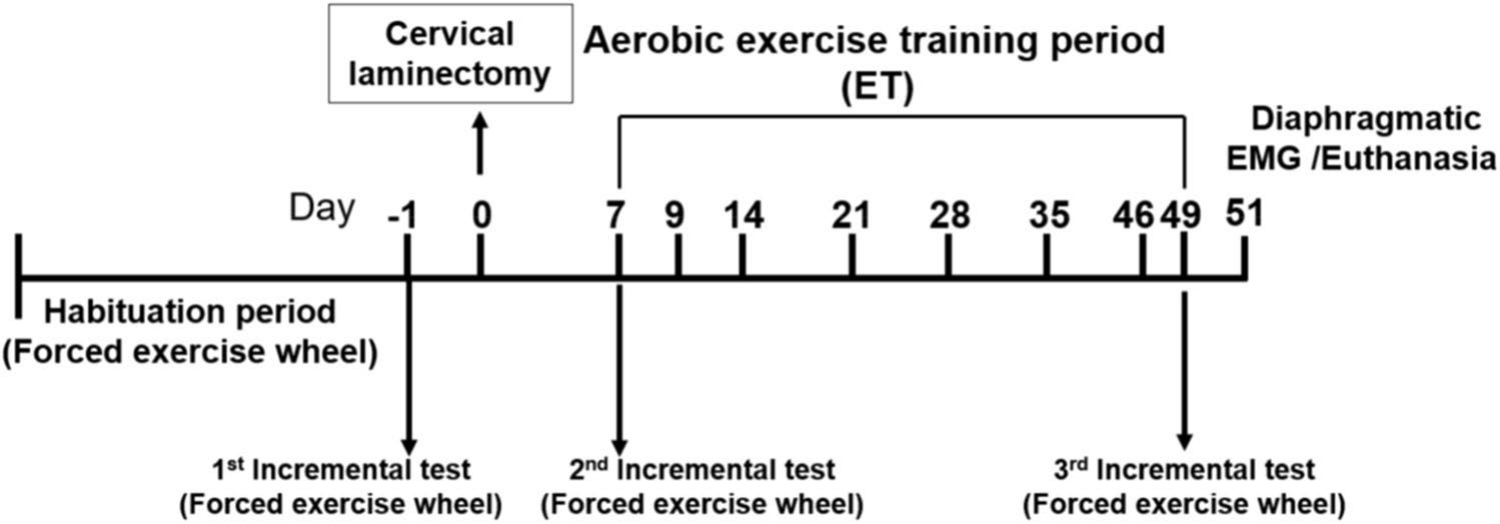
Experimental design throughout surgery and low intensity exercise training periods. After 1st incremental test on forced exercise wheel, animals were distributed into six experimental groups (3 sedentary and 3 trained of NAIVE, SHAM and SCI). SHAM and SCI groups underwent cervical laminectomy followed by a 1-week recovery. Next, all groups went through a 6-week aerobic exercise training on forced exercise wheel.

A total of 36 animals were familiarized with the forced exercise wheel system (Lafayette Inst., Lafayette, IN) during 1 week (6 m/min, 10 min each day). After the acclimatization period, all mice performed an incremental exercise test adapted to forced exercise wheel system (see Jesus et al*.*^31^) with increments of 2 m/min every 2 min, until exhaustion, defined as the moment when animals were unable to keep up the pace in the wheel or when animals voluntarily stopped running. A custom-made polystyrene barrier was developed to motivate the animal and keep it running. After a first incremental exercise test (before SCI surgery) the mice were randomly separated in 3 groups: NAIVE (i.e. without surgery; n = 12, 6 females and 6 males), SHAM (i.e. with surgery but without C2 hemisection; n = 12, 8 females and 4 males) and SCI (i.e. with C2 hemisection surgery; n = 12, 5 females and 7 males). Afterwards, each NAIVE, SHAM and SCI group was equally divided (n = 6 to each group) into sedentary (without training) or trained subgroups (SED or TR). No difference in incremental exercise test results was observed between sexes. A second incremental exercise test was performed 7 days after SCI/SHAM surgery to determine the intensity of the exercise protocol (60% from maximal achieved speed). At the end of the exercise protocol, sedentary and trained mice performed a third incremental test to evaluate the running performance at 49 days after the SCI/SHAM surgery (Fig. 10).

### Chronic C2 hemisection

Animals were anesthetized with isoflurane (5% in 100% O_2_) in a closed chamber. Anesthesia was maintained throughout the procedure (1.5–2% isoflurane in 100% O_2_) through a nose cone. Animals were placed on a heating pad. After careful shaving of the dorsal part of the animal’s neck, the skin and muscles above the first vertebrae were retracted. A C2 laminectomy and a durotomy were performed. The spinal cord was ventro-laterally hemisected caudal to the C2 dorsal roots by using microscissors followed by a micro-scalpel to ensure the sectioning of all remaining fibers. Sutures were performed to close the wounds and skin. SHAM mice underwent the same procedures without the hemisection. The isoflurane vaporizer was then turned off and mice received subcutaneous injections of analgesic (buprenorphine, 375 µg/kg: Buprecare from Axience) and antibiotics (40 mg/kg Trimethoprim and 200 mg/kg Sulfadoxin: Borgal 24%, from Virbac).

### Exercise training protocol paradigm

Exercise training was applied during 6 weeks (1 week after surgery) and consisted in having a mouse run on a forced exercise wheel system, at 60% of the maximal speed measured during the second incremental test (7 days after SCI surgery), 1 h per day and 5 days a week. The exercise training intensity was adapted from a protocol previously described by Ferreira et al*.*^30^*.* SED mice were kept in non-moving wheel for the entire duration of the exercise session.

### Electrophysiological recordings

Two days after the third incremental test (49 days after SCI surgery), anesthesia was induced using 5% isoflurane in 100% O_2_ and maintained throughout the experiment (2.5% in 21% O_2_) through a nose cone. As previously described by Michel-Flutot et al*. *^11^, animals were placed in supine position on a heating pad to maintain a physiological and constant body temperature. A laparotomy was performed to access the diaphragm. Both sides (ipsilateral and contralateral to the spinal cord lesion) of the diaphragm were tested for their activity (electromyography) using a handmade bipolar surface silver electrode on the muscle during spontaneous poïkilocapnic normoxic or transient mild asphyxia breathing (by occlusion of the animal nose for 15 s)^55^. Electromyographies were amplified (Model 1800; gain, 100; A-M Systems, Everett, WA, USA) and band pass-filtered (100 Hz to 10 kHz). The signals were digitized with an 8-channel Powerlab data acquisition device (Acquisition rate: 4 k/s; AD Instruments, Dunedin, New Zealand) connected to a computer and analyzed using LabChart 9 Pro software (AD Instruments, Dunedin, New Zealand). The bilateral diaphragmatic electromyographies were integrated (50 ms decay). At the end of the experiment, animals were humanly euthanized by CO_2_ and then intracardiac perfusion of heparinized 0.9% NaCl (10 mL) was performed for tissue harvesting. The C1-C3 segment of the spinal cord was dissected out and immediately placed overnight in cold 4% paraformaldehyde, then cryoprotected for 48 h in 30% sucrose (in 0.9% NaCl) and samples were stored in a freezer at − 80 °C. Forelimb (EDC) and hindlimb (TA and SOL) muscles from the ipsilateral side, and respiratory muscle (Ipsilateral and Contralateral DIA) were then dissected and immediately flash-frozen in isopentane (2-methylbutane) chilled with liquid nitrogen. After freezing, muscle samples were stored at − 80 °C for further processing.

### Immunofluorescence labeling of muscle tissue

All frozen skeletal muscles were placed in a Leica cryostat and transverse sections of 10 μm were performed. As previously described by Gill et al.^15^, sections were blocked for 45 min in 10% goat serum diluted in PBS 1X solution and incubated overnight at 4 °C in primary antibodies for the following Myosin heavy chain (MyHC) isoforms: MyHCSlow (BA-F8, 1:100 dilution; Developmental Studies Hybridoma Bank, Iowa City, IA), MyHC2A (SC-71, 1:100 dilution; Developmental Studies Hybridoma Bank), and Laminin (Sigma L9393, 1:200 dilution; Sigma-Aldrich, St. Louis, MO). Secondary antibodies were then incubated at a 1:2000 dilution, using Alexa Fluor Goat anti-Mouse IgG2b 647 (for MyHCSlow), Goat anti-Mouse IgG1 555 (for MyHC2A) and Goat anti-Rabbit 594 (for Laminin)^15^. CD 31 (PECAM-1, 1:100; Thermo Fisher Scientific, Inc., MA) antibody was incubated with Laminin antibody and Alexa Fluor Goat anti-Rat 488 and Goat anti-Rabbit 594 were applied at 1:2000 dilution.

### Muscle measurements

Fields within stained cross-sections were captured at 10 × magnification on a laser confocal microscope (Leica TCS SPE) capable of simultaneous imaging of multilabeled fluorescence. Images were captured in a 1024 × 1024 pixels array, with similar acquisition parameters across preparations. The composition of the different types of myofibers (scale bars = 200 µm) was measured in approximately 200 muscle fibers from each muscle in each animal and was analyzed using Fiji v1.52p (NIH Image du National Institutes of Health, USA). The same fibers selected for cross-sectional area measurements were also used to determine the proportion of fiber types in each muscle. Based on the staining pattern, all skeletal muscles fibers were classified as type I, type IIa, type IIx and/or IIb^15^. CD31-positive cells and the number of fibers were counted then, density and capillary-to-fiber ratios were calculated using Fiji v1.52p (NIH Image du National Institutes of Health, USA).

### Histological reconstitution of the extent of C2 injury

Longitudinal Sects. (30 μm thickness) were obtained with a Leica cryostat and the extent of injury was assessed after cresyl violet staining as previously described^11^. Slides were then scanned with an Aperio AT2 scanner (Leica, France) and a high-resolution picture of each slide was taken to evaluate the extent of the injury and reported on a stereotaxic transverse plane (C2). Each injury was then digitized and analyzed with Fiji v1.52p (NIH Image du National Institutes of Health, USA). The percentage of damaged tissue on the injured side was calculated by reference to a complete hemisection (which is 100% of the hemicord).

### Data processing and statistical analysis

For diaphragm electromyography recordings, the amplitude and duration of minimally 5 double integrated bursts during spontaneous breathing and mild asphyxia were calculated for each animal from both side of the diaphragm with LabChart 8.1.9 Pro software (AD Instruments) www.adinstruments.com Student’s t test was performed between the different groups for extent of injury evaluation or between the different post-lesioned time groups of the same condition. Paired t-test was used to compare: (1) the animal running capacity through incremental test at different time points (before SCI, 7 days after SCI and 49 days after SCI), (2) contralateral and ipsilateral diaphragmatic electromyograms on the same animal, and (3) immunofluorescence stains between tissues from the same animal. Two-way ANOVA (Bonferroni post hoc test) was used to compare training (SED and TR) and group (NAIVE, SHAM and SCI group). All data were presented as mean ± SD and statistics were considered significant when *p* < 0.05. SigmaPlot version 12.5, from Systat Software, Inc., San Jose California USA, www.systatsoftware.com was used for all analyses.

## Supplementary Information

Supplementary Information.
