# Supplementary material for: Effects of aerobic exercise training on muscle plasticity in a mouse model of cervical spinal cord injury
Source: Sci Rep. 2021 Jan 8;11:112. doi: 10.1038/s41598-020-80478-9 (PMC7794462; doi:10.1038/s41598-020-80478-9)
Supplement: Supplementary file 1 — Supplementary Information. [file 41598_2020_80478_MOESM1_ESM.docx]

**Supplemental data**

**Effects of aerobic exercise training on muscle plasticity in a mouse model of cervical spinal cord injury**

*Running title:* **Exercise following spinal cord injury**

Authors and Affiliations:

Isley Jesus^1^, Pauline Michel-Flutot^1^, Therese B. Deramaudt^1^, Alexia Paucard^1^, Valentin Vanhee^1^, Stéphane Vinit^1^ and Marcel Bonay^1*^

^1^Université Paris-Saclay, UVSQ, Inserm, END-ICAP, 78000 Versailles, France

*Corresponding author: Marcel Bonay, MD, PhD

E-mail : marcel.bonay@aphp.fr

**
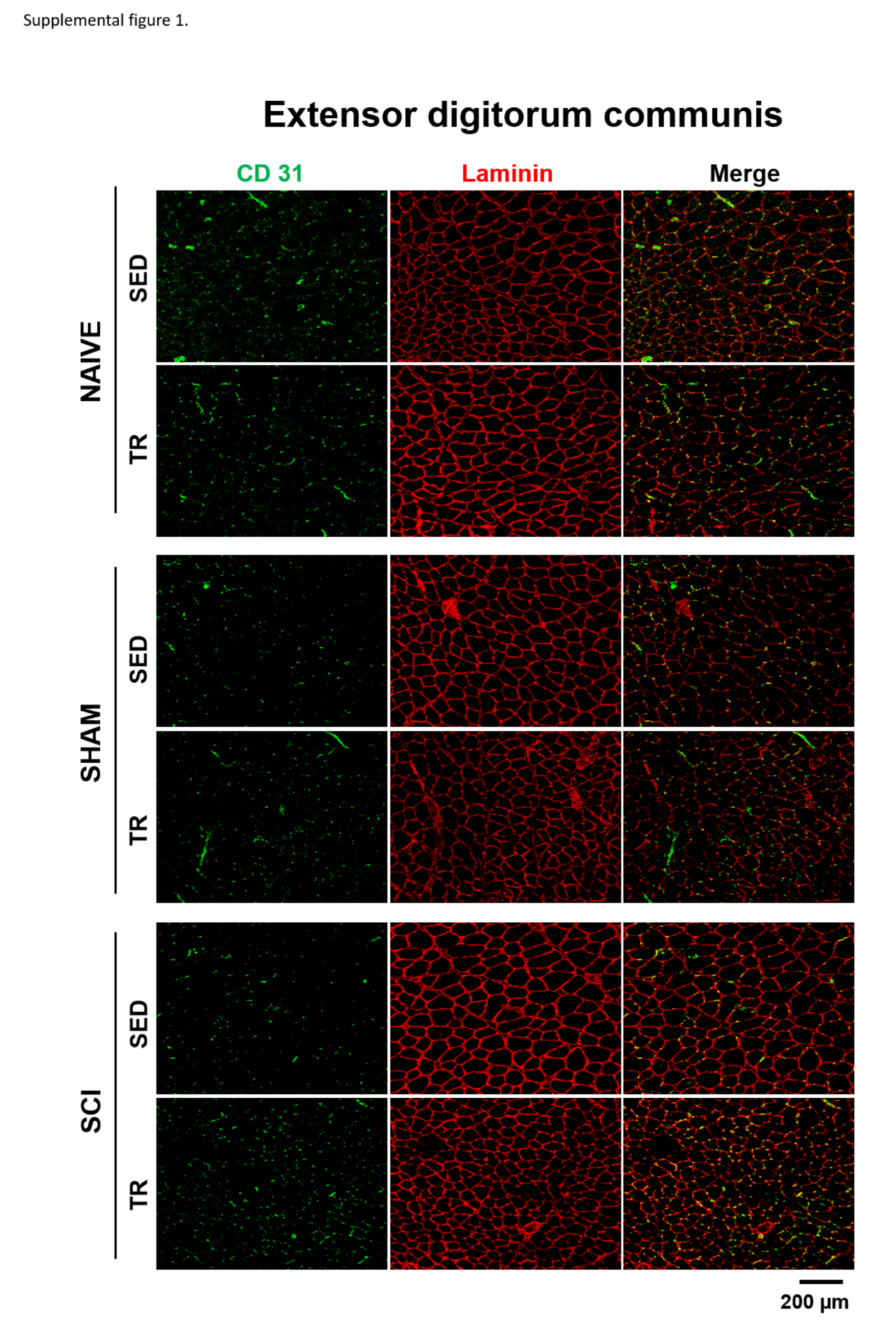
**


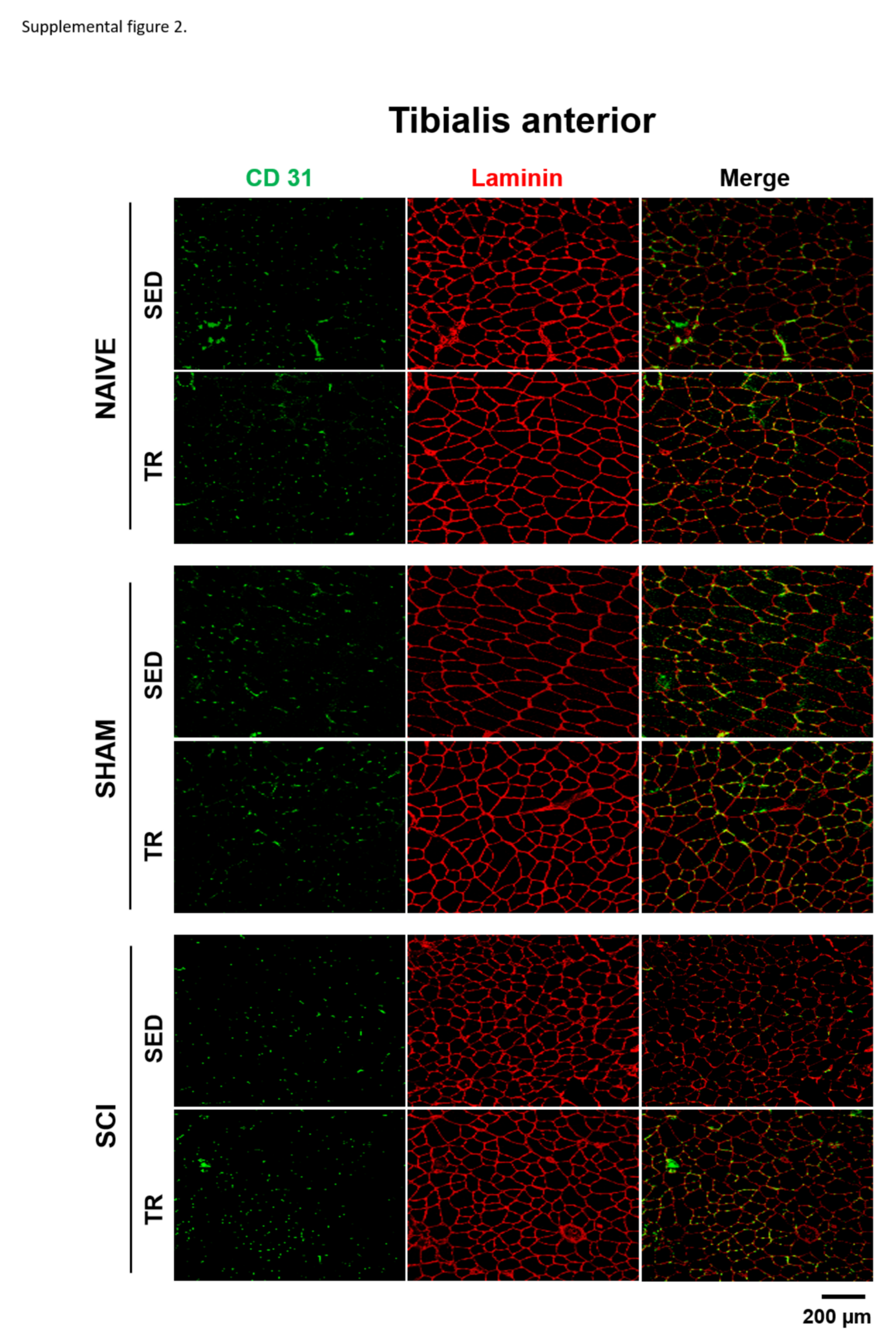


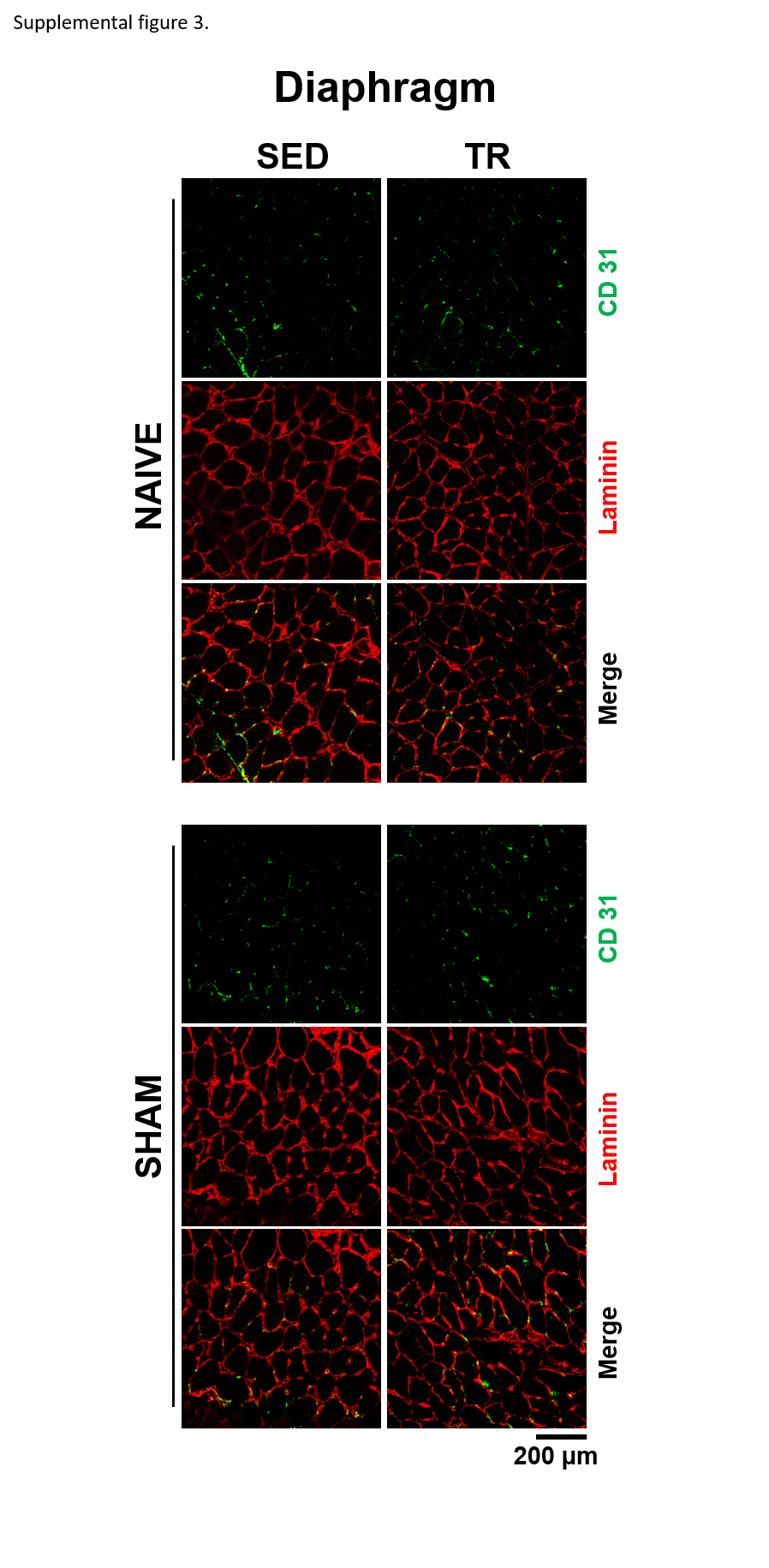


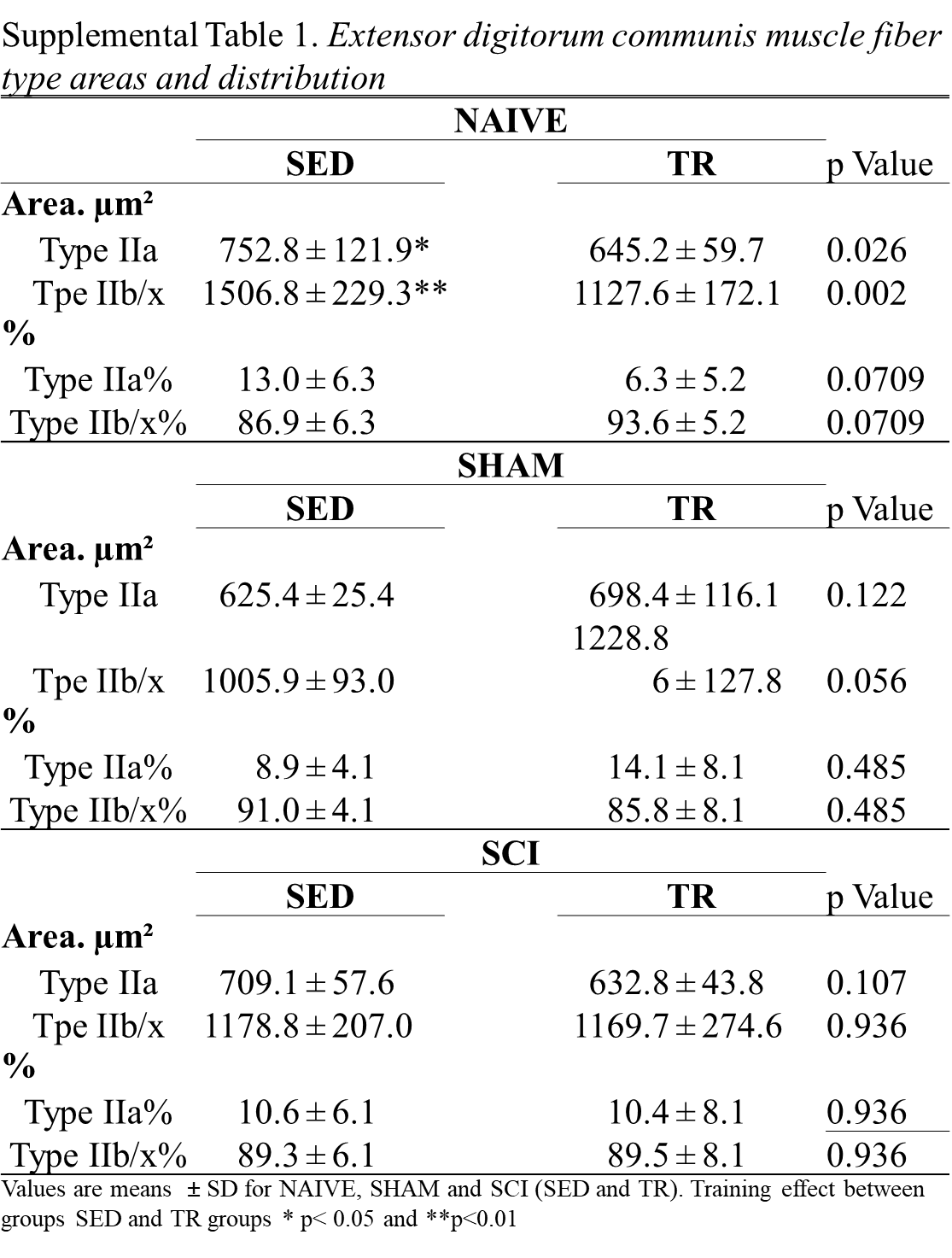

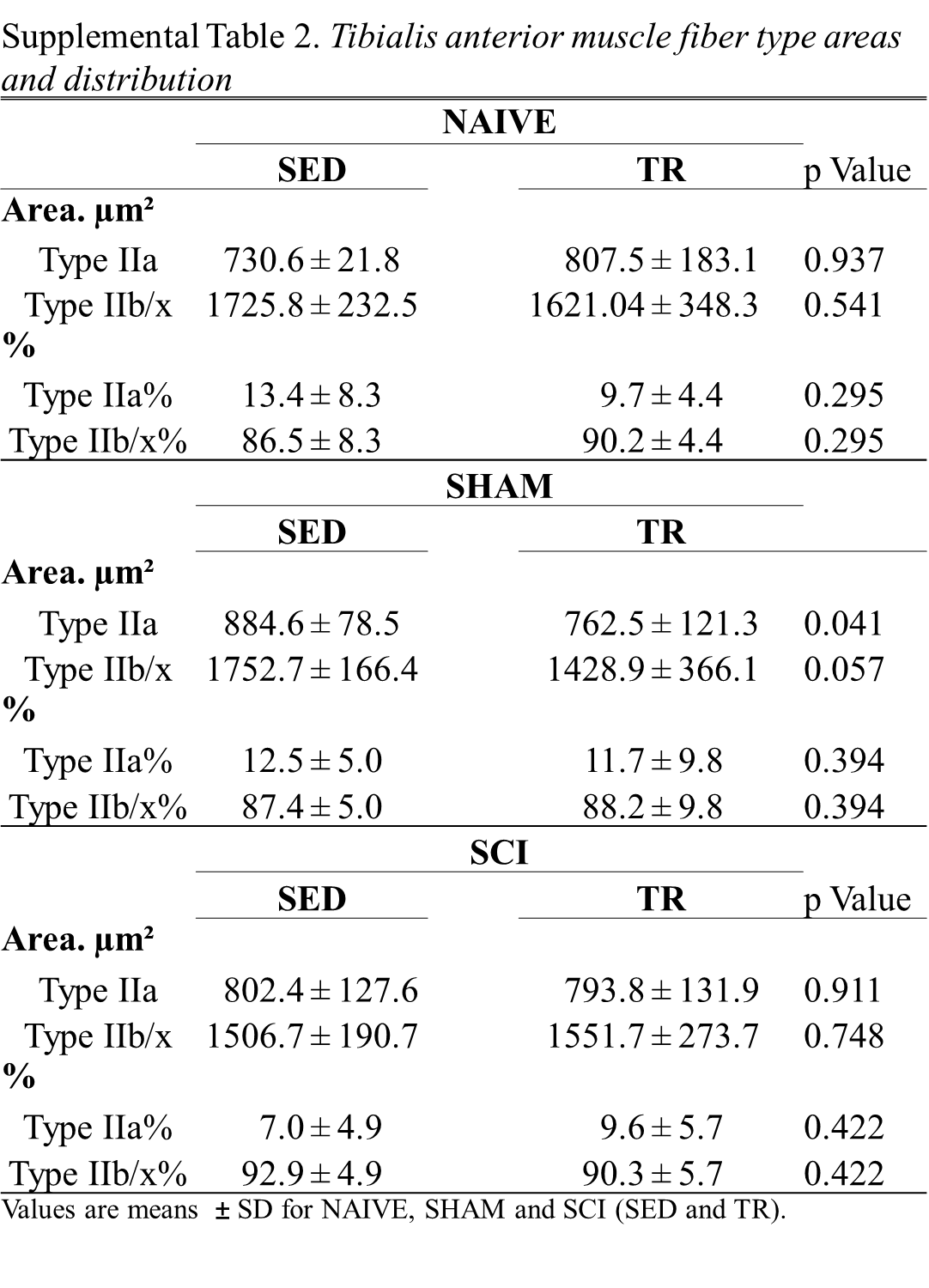


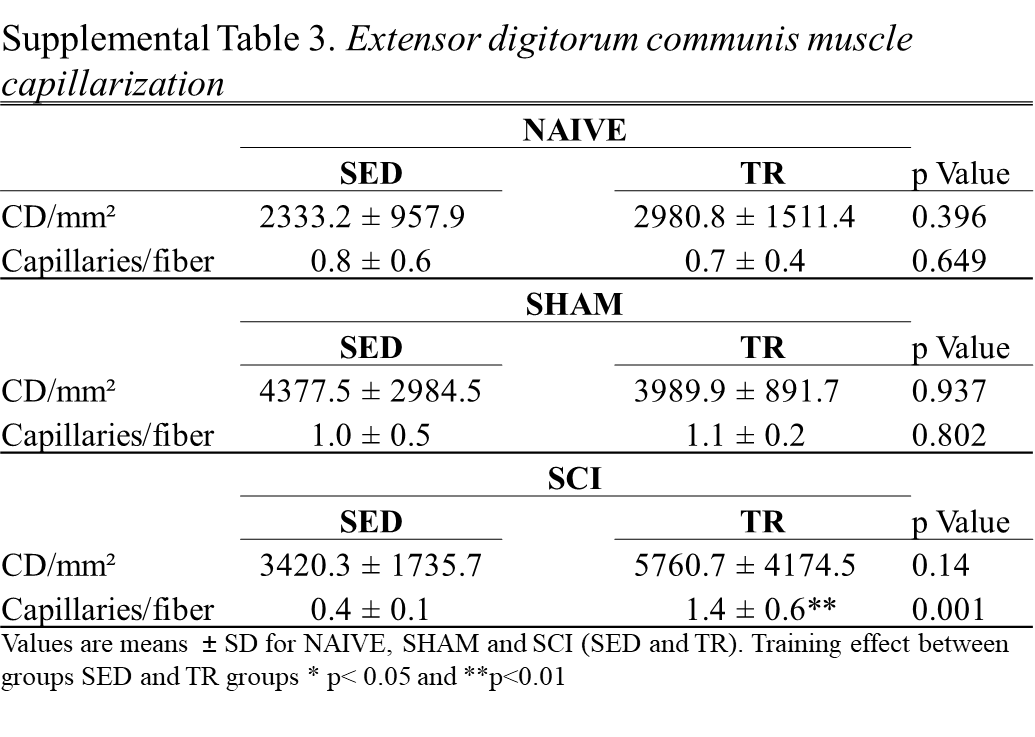


**
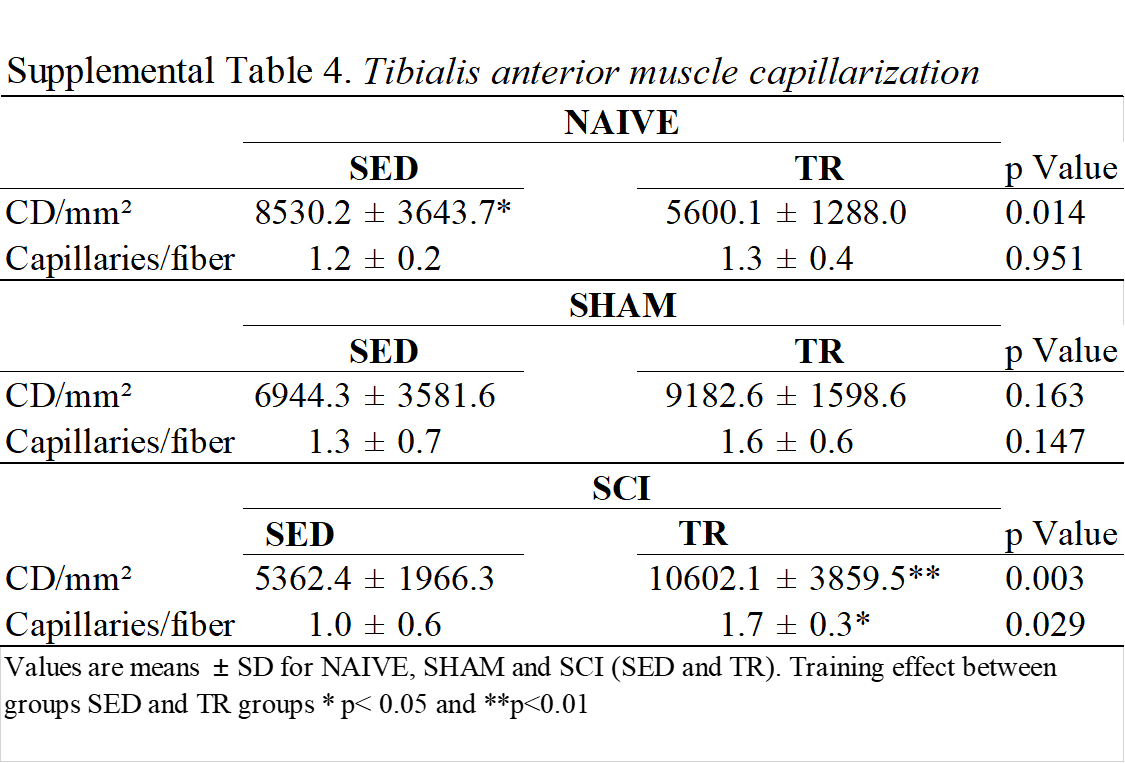
**

**SUPPLEMENTAL FIGURES**

**Supplemental figure 1. Representative images of CD31 expression and number of fibers in the skeletal muscles.** Cryosections were immunostained to visualize CD31 (green) co-labeled with an anti-Laminin antibody (in red) in Extensor digitorum communis (EDC). Scale bar=200 μm.

**Supplemental figure 2. Representative images of CD31 expression and number of fibers in the skeletal muscles.** Cryosections were immunostained to visualize CD31 (green) co-labeled with an anti-Laminin antibody (in red) in Tibialis anterior (TA). Scale bar=200 μm.

**Supplemental figure 3. Representative images of fiber type composition in respiratory muscle. A** Diaphragmatic muscle cryosections were immunostained to visualize Myosin heavy chain (MyHC) isoforms: MyHCSlow (type I in blue), MyHC2A (type IIa in green) co-labeled with an anti-laminin antibody to visualize myofiber boundaries (type IIb/x corresponding to unlabeled fibers) in NAIVE and SHAM groups (SED and TR). Scale bar=200 μm.

**SUPPLEMENTAL TABLES**

**Supplemental Table 1**. **Extensor digitorum communis muscle fiber type areas and distribution**. Values are means ± SD for NAIVE, SHAM and SCI (SED and TR). Training effect between groups SED and TR groups * p< 0.05 and **p<0.01.

**Supplemental Table 2. Tibialis anterior muscle fiber type areas and distribution**. Values are means ± SD for NAIVE, SHAM and SCI (SED and TR).

**Supplemental Table 3**. **Extensor digitorum communis muscle capillarization**. Values are means ± SD for NAIVE, SHAM and SCI (SED and TR). Training effect between groups SED and TR groups * p< 0.05 and **p<0.01.

**Supplemental Table 4**. **Tibialis anterior muscle capillarization**. Values are means ± SD for NAIVE, SHAM and SCI (SED and TR). Training effect between groups SED and TR groups * p< 0.05 and **p<0.01.
